# Pull-through method for central venous catheter placement in a case of cadaveric donor small bowel retransplantation

**DOI:** 10.1186/s40981-021-00411-5

**Published:** 2021-01-12

**Authors:** Kayo Kimura, Shuji Kawamoto, Shinichi Kai, Tomoharu Tanaka, Kazuhiko Fukuda

**Affiliations:** Department of Anesthesia, Kyoto University Hospital, 54, Shogoinkawahara-cho, Sakyo-ku, Kyoto, 606-8507 Japan

**Keywords:** Cadaveric donor small bowel transplantation, Retransplantation, Difficulty securing the central venous route, Pull-through method

To the Editor,

Small bowel transplantation is indicated in patients with short bowel syndrome or irreversible small bowel insufficiency who have progressive liver dysfunction or difficulty with securing the central venous route after long-term intravenous feeding [1, 2]. We report the fifth case of cadaveric donor small bowel retransplantation in Japan [3], in which a central venous route was secured using the pull-through method.

The patient was a 22-year-old female with a height of 131.3 cm and a weight of 30.9 kg. Intravenous feeding was started due to small bowel insufficiency of unknown cause after birth. The catheter had to be changed due to frequent infections, and it became increasingly difficult to secure the central venous route. Her first cadaveric donor small bowel transplantation was performed at age of 13. The transplanted small bowel gradually became dysfunctional due to chronic rejection, and intravenous feeding was planned to be reintroduced, but the central venous route was already depleted. A Broviac catheter was inserted percutaneously and transhepatically through the left hepatic vein. Cadaveric donor small bowel retransplantation was determined to be indicated.

The establishment of a central venous route was essential for her perioperative management. Contrast-enhanced computed tomography showed slight patency in the left internal jugular vein. We punctured the left internal jugular vein, and a guidewire was advanced with ultrasound guidance under local anesthesia. However, there was a stenosis in the innominate vein, and the catheter could not be advanced beyond that. We decided to try the pull-through method, which is useful for inserting a catheter into a narrow or tortuous vessel. By using this method, sufficient tension can be applied to the guidewire, which allows the catheter to be stably advanced to a target site [4, 5]. In this case, the snare wire was advanced retrograde through the right inferior hepatic vein with ultrasound guidance under local anesthesia without sedation and passed through the innominate vein stenosis. The guidewire inserted from the left internal jugular vein was captured by the snare wire and withdrawn from the right inferior hepatic vein outside the body. The guidewire was firmly tensioned from the cephalad and caudal sides, and the catheter could be placed through the left internal jugular vein (Fig. 1). Percutaneous transhepatic tract embolization was performed after the procedure. This catheter and the percutaneous transhepatic Broviac catheter were used as venous routes during the perioperative period (Fig. 2).

**Fig. 1:**
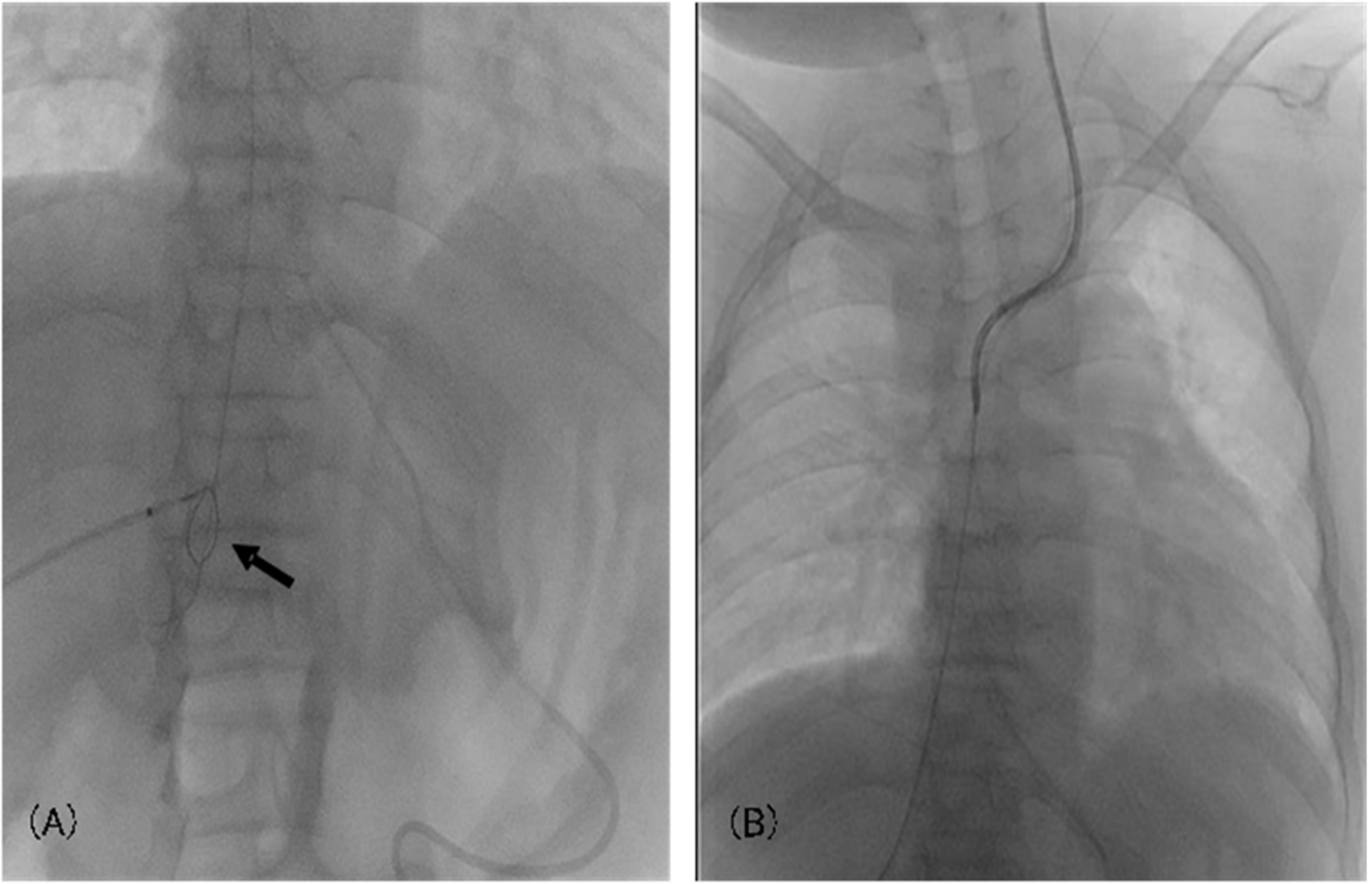
A central venous catheter placement using the pull-through method. A snare wire (arrow) was used to capture the inserted wire from the left internal jugular vein and pulled out of the right inferior hepatic vein outside the body (**a**). A central venous catheter was inserted through the left internal jugular vein (**b**)

**Fig. 2:**
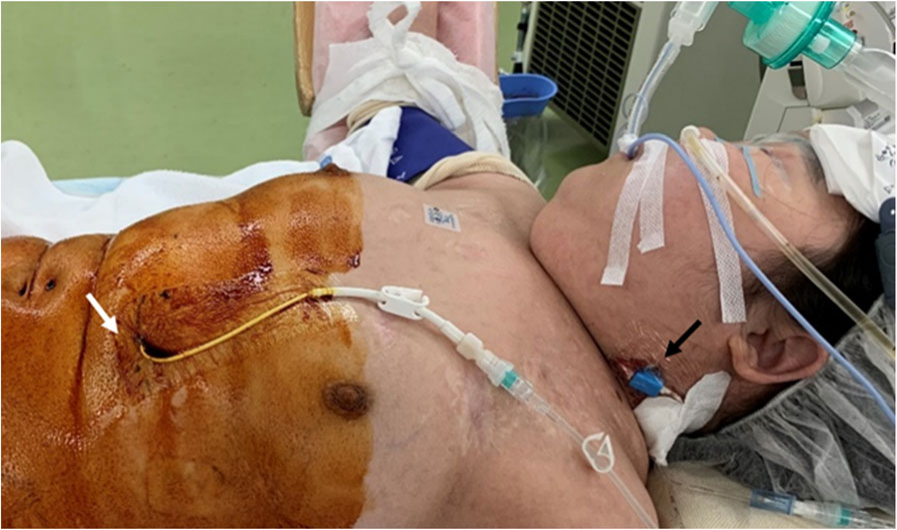
A photograph after the induction of anesthesia. A Broviac catheter is placed in the left hepatic vein (white arrow), and a central venous catheter is placed in the left internal jugular vein (black arrow)

Central venous routes from the internal thoracic vein, intercostal vein, inferior abdominal wall vein, hepatic vein, and gonadal vein have been used in cases of obstruction or stenosis of major vessels. However, most such reports have been in pediatric cases [6–8]. A hepatic venous approach by ultrasound-guided percutaneous transhepatic puncture has also been reported [9]. The technique is relatively safe, like percutaneous transhepatic biliary drainage, and the risk of bleeding is low; however, general anesthesia is required for the placement. In contrast, securing the central venous route via the internal jugular vein using the pull-through method can be performed under local anesthesia. We suggest that this is a useful option in cases where the central venous route is difficult to secure.

## Data Availability

All data generated or analyzed in this study are included in this article.
